# Novel dental implant modifications with two-staged double benefits for preventing infection and promoting osseointegration *in vivo* and *in vitro*

**DOI:** 10.1016/j.bioactmat.2021.04.041

**Published:** 2021-05-15

**Authors:** Xiaoyu Huang, Yang Ge, Bina Yang, Qi Han, Wen Zhou, Jingou Liang, Mingyun Li, Xian Peng, Biao Ren, Bangcheng Yang, Michael D. Weir, Qiang Guo, Haohao Wang, Xinxuan Zhou, Xugang Lu, Thomas W. Oates, Hockin H.K. Xu, Dongmei Deng, Xuedong Zhou, Lei Cheng

**Affiliations:** a State Key Laboratory of Oral Diseases, West China Hospital of Stomatology, National Clinical Research Center for Oral Diseases, Sichuan University, Chengdu, 610041, China; b Department of Operative Dentistry and Endodontics, West China School of Stomatology, Sichuan University, Chengdu, 610041, China; c Stomatological Hospital, Southern Medical University, Guangzhou, 510280, China; d Department of Pathology, West China School of Stomatology, Sichuan University, Chengdu, 610041, China; e Department of Pediatrics, West China School of Stomatology, Sichuan University, Chengdu, 610041, China; f Department of Advanced Oral Sciences and Therapeutics, University of Maryland School of Dentistry, Baltimore, MD, 21201, USA; g Department of Preventive Dentistry, Academic Center for Dentistry Amsterdam (ACTA), University of Amsterdam and VU University Amsterdam, Amsterdam, the Netherlands; h National Engineering Research Center for Biomaterials, Sichuan University, Chengdu, 610041, China

**Keywords:** Peri-implantitis, Dimethylaminododecyl methacrylate, Animal model, Osseointegration, Bioactive and therapeutic

## Abstract

Peri-implantitis are a major problem causing implant failure these days. Accordingly, anti-infection during the early stage and subsequent promotion of osseointegration are two main key factors to solve this issue. Micro-arc oxidation (MAO) treatment is a way to form an oxidation film on the surface of metallic materials. The method shows good osteogenic properties but weak antibacterial effect. Therefore, we developed combined strategies to combat severe peri-implantitis, which included the use of a novel compound, PD, comprising dendrimers poly(amidoamine) (PAMAM) loading dimethylaminododecyl methacrylate (DMADDM) as well as MAO treatment. Here, we explored the chemical properties of the novel compound PD, and proved that this compound was successfully synthesized, with the loading efficiency and encapsulation efficiency of 23.91% and 31.42%, respectively. We further report the two-stage double benefits capability of PD + MAO: (1) in the first stage, PD + MAO could decrease the adherence and development of biofilms by releasing DMADDM in the highly infected first stage after implant surgery both *in vitro* and *in vivo*; (2) in the second stage, PD + MAO indicated mighty anti-infection and osteoconductive characteristics in a rat model of peri-implantitis *in vivo*. This study first reports the two-staged, double benefits of PD + MAO, and demonstrates its potential in clinical applications for inhibiting peri-implantitis, especially in patients with severe infection risk.

## Highlights

• PAMAM-DMADDM shows toxicity reduction and osteoconductive properties *in vitro.*
• Micro-arc oxidation treatment shows osteoconductive but weak antibacterial effect.
• PAMAM-DMADDM + Micro-arc oxidation treatment shows multifunctional properties.

## Graphical abstract

**Figure:**
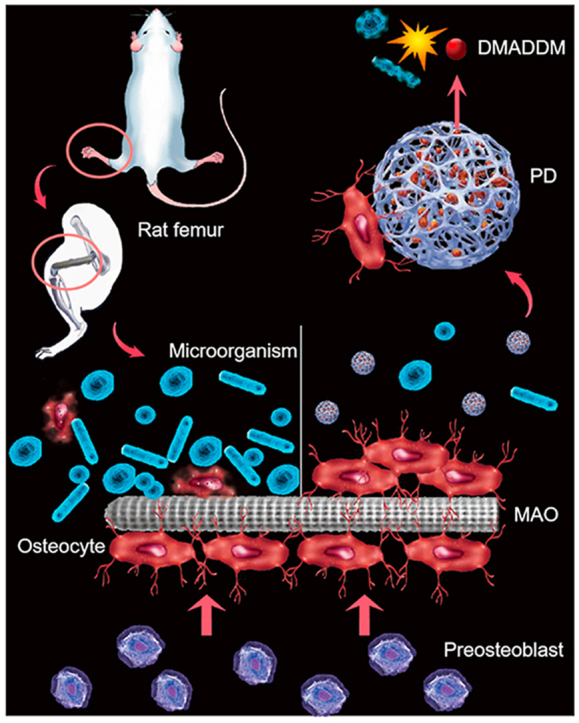

## 1 Introduction

Millions of implants are used each year in medical care, and most of these are colonized by bacteria and fungi, which have become the focus of peri-implantitis [1]. Study shows that the occurrence of peri-implantitis is about 15.3% of patients as well as 9.2% of implants [2]. Each infectious implant should cost more than 332.87 euros (€332.87) each year compared to the healthy ones [3]. Moreover, with the implants are widely used, the number of infectious cases continues to increase [4]. Microbial adhesion to the surface of implant and following biofilm development at the implantation site is one of the major pathogenesis of peri-implantitis [5,6]. Both *Staphylococcus aureus* and *Candida albicans* are detected in mixed species implant infections [7,8] and usually cause severe infections [9]. Traditional antibiotic treatments use intravenous antibiotics and local antibiotic application, both of which have limited efficacy [10], whereas antibiotic resistance in microorganism continues to grow because of overusing antibiotics [11]. Highly virulent bacteria and fungi such as *S. aureus* and *C. albicans*, respectively, will adhere to the implant surface and development biofilms, especially in cases where the immune system is compromised [12,13]. Accordingly, prevention of infection is important in such situations [14].

Microbial adhesion usually initiated in the early stage of treatment, which is defined as the first four weeks after implant surgery [[15], [16], [17]]. Aggressive inflammation and long-term infection lead to osteolysis around dental implants, and therefore, weaken osseointegration in the implantation site, which is another major factor causing implant failure [11,18]. Decreasing the initial microbial adhesion to the site of implant is important to prevent against early infection in the first stage [18]. After the antibacterial stage, the second stage post-operation warrants a strong osseointegration capability and a sustained antibacterial activity [19].

There are two main strategies to solve the problem of peri-implantitis: implant material surface modification and local drug treatment [11,17]. Development of anti-infective biomaterials is a major preventive method these days [[20], [21], [22]]. Ideal strategies should have advantages such as favorable biocompatibility, anti-biofilm properties, and osseointegration promotion. During recent years, novel dental implant materials have been developed to control peri-implantitis [19,23]. However, seldom dental implants were designed to investigate all the three problems mentioned above, for implant surfaces which support osteointegration may also favor colonization of bacterial cell, or the antibacterial implants may also impair the cell proliferation and osteointegration [11,25]. In our previous studies, we reported novel implant materials, which had antibacterial and osteogenic properties, via micro-arc oxidation (MAO) treatment [26,27]. However, for immunocompromised individuals and those with highly virulent microorganism infections, local drug treatment is needed in the high-risk stage. Dimethylaminododecyl methacrylate (DMADDM) is a kind of quaternary ammonium methacrylate (QAM) [28]. DMADDM has previously been proved to be a strong antibacterial agent against a broad spectrum of microbial species [[29], [30], [31]]. However, cytotoxicity is a major concern when using DMADDM monomer. Dendrimer poly(amidoamine) (PAMAM) is a type of polymer which can simulate the acid non-collagen proteins (NCPs). The NCPs can help to induce or adjust the crystallization and nucleation of hydroxyapatite, which forms hard tissues [32]. Moreover, the cavity of dendrimer shows function of a good drug carrier, usually used to synthesis dual function compounds of hard tissue regeneration as well as anti-microbial effects [33]. PAMAM has been reported to increase cell adhesion, causing enhanced cellular spreading and actin organization [34]. Therefore, DMADDM-loading PAMAM (PAMAM-DMADDM, PD) may combine the advantages of both molecules to have the antibacterial effects and decrease the toxicity of DMADDM through the slow release during the first stage as well as biomimetic mineralization promotion. After the effective control of infection in the first stage, MAO implants can provide a sustained anti-bacterial effect and continue to promote osseointegration. Therefore, the combined strategies of MAO and PD appeared to be suitable for combating peri-implantitis.

The present study reports a novel biomaterial PD and its combination with MAO to combat peri-implantitis at two stages. PD and MAO both have antibacterial and osseointegration-promoting effects. In this study, a highly infected rat model, which provides a foundation for clinical application, was used to test the multifunction of PD and MAO. The study indicated that local treatment with PD provides a strong antibacterial activity at the high-risk first stage of initial four weeks especially for individuals with severe infections or immunocompromised individuals. In the second stage, MAO implants will mainly have an antibacterial effect and promote osseointegration. Antimicrobial characteristics, anti-infection abilities, and osteogenesis characteristics of PD and MAO treatment *in vitro* and *in vivo* have been evaluated in the study.

## 2 Materials and Methods

### 2.1 Preparation of titanium samples

Titanium discs were prepared in the size of 6 mm in diameter and 1 mm in thickness, and titanium rods were prepared in the size of 1.5 mm in diameter and 20 mm in length. The novel MAO implant was made by an anodizing device. The samples were immersed into a 1 M H_2_SO_4_ solution. Subsequently, a 70 V DC voltage for 1 min was applied as previously reported [27,35]. Sandblasting and acid etching (SLA) and plasma spraying with hydroxyapatite (HA) are dental implant treatments for commercial use. In brief, the SLA was first processed by sandblasting treatment by Al_2_O_3_ particles, and etched in acid solution (sulfuric acid, hydrochloric acid: H ₂ O = 1:1:2); the HA was first sandblasted and then plasma sprayed with about 30 μm of hydroxyapatite; the machined technique was mechanically polished in sequence with grit SiC paper (#180, #400, #800, #1200). In this experiment, HA, SLA, and machined titanium act as controls. All discs and rods were sterilized in an ethylene oxide sterilizer (AnproleneAN 74i; Andersen, Haw River, NC, USA).

### 2.2 PD synthesis

The same weight (1 g) of PAMAM and DMADDM were mixed in ethanol and stirred at room temperature for 1 h then 55 °C for 3 h. The mixed solution was added to a 1000D dialysis bag, and rotate dialysis for 2 days. Following this, the mixed solution was freeze dried. Then collecting the solid powder and storing at −20 °C for later use. Subsequently, ^1^H NMR spectroscopy, particle size testing, Zeta potential testing, loading efficiency evaluation, and release experiment *in vitro*, as well as biocompatibility testing *in vivo* and *in vitro* were performed.

The detailed methods of testing the ^1^H NMR spectroscopy, particle size, and Zeta potential were showed in the Supplementary materials. To calculate the loading efficiency of PD, the known concentration of DMADDM was diluted in a gradient, and ultraviolet absorbance measurement (Shimadzu, Japan) at a wavelength of 220 nm was used to determine the standard curve. Then the dialyzed fluid was collected, diluted 160 times, and the concentration of non-carried DMADDM was detected via ultraviolet absorbance measurement (Shimadzu, Japan) at a wavelength of 220 nm. The loading efficiency and encapsulation efficiency of PD was calculated using the following formula:

Loading efficiency = M_D_/(M_P_ + M_D_) [36]

Encapsulation efficiency = M_D_/M_TD_ [37]

M_D_ is the quantity of DMADDM that was loaded within PAMAM. M_P_ is the quality of PAMAM. M_TD_ is the quality of DMADDM added in the beginning. Ultraviolet absorbance measurement was used to determine the concentration of DMADDM within PD.

For the releasing experiment, 780 mg PD was added to dialysis tubing and the release experiment was conducted in 100 mL ethanol. Then, 1 mL of the solution was collected for later detection and replaced with fresh ethanol at 1, 2, 3, 4, 5, 6, 12, 24, 36, 48, 72, 96, 168 (1 week), 336 (2 weeks), and 504 h (3 weeks), respectively. The concentrations of the DMADDM were tested by the method mentioned above.

### 2.3 *In vitro* experiment

Antibacterial experiment: Titanium was added to a 24-well plate, then added 1 mL RPMI1640 medium (GlutaMAX™ Supplement, Thermo Fisher Scientific, USA) to each well. PD solution at a mass fraction of 0.25 mg/mL and 0.5 mg/mL, or PBS as a control, was subsequently added to the wells. *S. aureus* and *C. albicans* were diluted to 1 × 10^6^ CFU/mL in the medium [38]. After 24 h of anerobic incubation, MTT (3-(4,5-dimethyl-thiazol-2-yl)-2,5-diphenyltetrazolium bromide) assay which was used to determine the metabolic activity of biofilm, crystal violet assay that was to detect biofilm accumulation, SEM, live and dead staining, and CFU assay were performed. The methods of multi-species biofilm formation, MTT assay, crystal violet assay, SEM, live and dead staining and CFU assay could be seen in the Supplementary materials.

The effect of PD on preosteoblasts: MC3T3-E1 mouse preosteoblastic cells (Sigma, USA) were inoculated in the α-MEM medium containing 10% fetal bovine serum (primary medium; PM). The cells were seeded at a density of 1 × 10^5^ cells/mL. All groups were incubated in an incubator at a constant temperature of 37 °C under 5% CO_2_ for 24 h. Then the medium was refreshed with several kinds of medium: osteoinductive medium (OM), PM, OM + 0.25 mg/mL PD, OM + 0.5 mg/mL PD and OM + 0.5 mg/mL PAMAM. After incubating for 24 h, all the groups except for the PM group were refreshed with OM, while the medium in PM group was refreshed with PM. The medium was changed the next day and the cells were incubated for 4, 7, 14, and 21 days [19]. MTT, ALP, and qPCR were performed, which could be seen in Supplementary materials.

### 2.4 *In vivo* experiment

All the animal experiments were followed by the ARRIVE guidelines 2.0, the details of checklist could be seen in the Supplementary materials Table S2 [39]. A femur implant rat model was used to assess the biosafety of MAO + PD, which was approved by West China Hospital of Stomatology Ethics Committee (WCHSIRB-D-2020-433). Twenty-five Sprague-Dawley (female, 12 weeks old) rats were randomly divided into five groups, which was followed by the previous study [19], control group, 0.25 mg/mL PD group, 0.5 mg/mL PD group, 0.5 mg/mL PAMAM group, and 0.5 mg/mL DMADDM group. Before implant surgery, the sterile MAO implants were immersed in PBS, 0.25 mg/mL PD, 0.5 mg/mL PD, 0.5 mg/mL PAMAM, and 0.5 mg/mL DMADDM respectively for 40min, and then dried in a sterile environment. After general anesthesia, the Ti rods were implanted into the left femurs as showed before [40]. The body weight of rats was recorded every week. For the assessment of the inflammation, the tail venous blood of the rats was collected at 3 and 6 weeks after implant surgery, and the neutrophils and hemoglobin were detected (n = 5). To evaluate the level of bone destruction, the rats were sacrificed at 6 weeks, and the micro-CT was used to scan the femurs. Three-dimensional images were collected and Tb.Th and BV/TV were analyzed (n = 5). Then, the histopathological assessment was carried out for the femurs without implants. The detailed information of micro-CT and histopathological assessment could be seen in Supplementary materials.

The anti-infection and osteointegration effect of MAO + PD were assessed by a highly infected rat model. Fourty-five Sprague-Dawley (female, 12 weeks old) rats were divided into three groups, MAO + *S. aureus*-*C. albicans* + 0.5 mg/mL PD, MAO + *S. aureus*-*C. albicans* + 0.25 mg/mL PD, and MAO + *S. aureus*-*C. albicans* + PBS, which were abbreviated as 0.5 mg/mL PD, 0.25 mg/mL PD, and PBS groups. Briefly, three kinds of solution were prepared at first, the *C. albicans* + *S. aureus* + PBS*, C. albicans* + *S. aureus* + 0.25 mg/mL PD, and *C. albicans* + *S. aureus* + 0.5 mg/mL PD, and both the *C. albicans* and *S. aureus* were at a concentration of 10^6^: 10^6^ CFU/mL. Then, MAO Ti rods were immersed in the solutions for 40min, dried in a sterile environment. Subsequently, after the rats were anesthetized, the Ti rods were implanted into the left femurs. For assessment of the cortical bone destruction of femurs, X-ray imaging was used after 1 day and 3 weeks of the implantation (n = 5). And the radiographic scores were evaluated according to the X-ray imaging (n = 5), the details of scoring were showed in previous study and the group names could not be seen when scoring [40]. For the gross pathology scoring and the counting of bacteria in the bone tissue, sacrifice the rats and collect the femurs in sterile conditions at 3 weeks (n = 5), the details of scoring was showed in previous study and the group names could not be seen when scoring [19,41]. To evaluate the level of bone destruction, five rats were sacrificed in each group, and the micro-CT was used to scan the femurs at 3 weeks and 6 weeks respectively (n = 5). Three-dimensional images were collected and Tb.Th and BV/TV were analyzed. Then, the histopathological assessment was carried out for the femurs without implants (n = 5). The details of Micro-CT and the histopathological assessment were showed in Supplementary materials. All the animal experiment were done in a sterile environment. Besides, the cages were kept in a *SPF*-grade laboratory animal room, and the water, food and bedding were changed every other day.

### 2.5 Statistical analyses

SPSS, version 22.0 (SPSS Inc., Chicago, IL, USA) was used to perform the statistical analysis. One-way analyses of variance (ANOVA) were performed to detect the significant effects of the variables at a p-value of 0.05.

## 3 Results and discussion

A very large percentage of implant-related infections are caused by *staphylococci* (approximately two-third), and most of them are by the highly virulent pathogen *S. aureus,* which accounts for approximately 35.5% infections [[42], [43], [44]]. *C. albicans* is a major fungal species in implant-related infections [38]. *S. aureus* and *C. albicans* are also often detected together in peri-implantitis [45]. The two species usually cause severe infections given their enhanced resistance to antimicrobials, strong surface colonization ability, and critical biological differences [9]. Biofilms derived by *C. albicans* and *S. aureus* are a relatively mature model for studying peri-implantitis; in this study, we evaluated the effect of MAO using this severe infection model [38,46,47].

The antibacterial effect of MAO implant material was observed via scanning electron microscopy (SEM), MTT assay, and colony-forming unit (CFU) assay. SEM showed that the amount of *C. albicans* was obviously reduced on MAO, but the amount of *S. aureus* did not change compared with the other three kinds of titanium discs (Supplementary Fig. 1ABC&D). Moreover, MTT analysis indicated that the metabolism of the biofilm on MAO surface decreased significantly (p < 0.05, Supplementary Fig. 1E). Furthermore, the CFU counts showed that MAO inhibited the adhesion of *C. albicans* significantly (p＜0.05, Supplementary Fig. 1F), but the adhesion of *S. aureus* did not change (Supplementary Fig. 1G).

MAO titanium has been reported to have good osteogenic characteristics [26], resistance to saliva biofilm bio-aging [48], and antibacterial characteristics because of its uniform surface compared with clinical commercial titanium implants such as sandblasting and acid etching (SLA) and plasma-sprayed hydroxyapatite (HA)implants [27]. It is a promising dental material, but when it comes to highly virulent complex biofilms such as those formed by *S. aureus* and *C. albicans*, the results have shown that the antibacterial effect of MAO is not strong enough to combat severe peri-implantitis. However, MAO titanium showed some antibacterial ability for inhibiting *C. albicans* (Supplementary Fig. 1F), which could maintain antibacterial ability after the high-risk first stage. Therefore, we developed a novel local drug PAMAM-DMADDM (PD) combined with MAO to combat peri-implantitis.

### 3.1 Characteristics of PD

#### 3.1.1 Material characterization

**Fig. 1:**
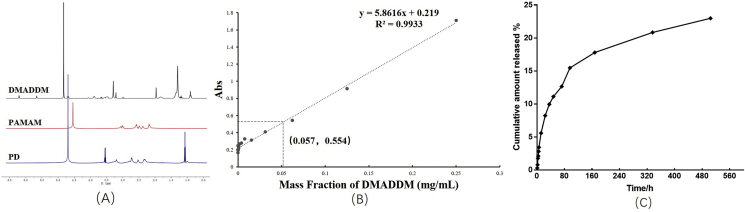
The structure and physical and chemical properties of the novel compound PD. (A) ^1^H-NMR; (B) Loading rate of DMADDM; (C) Release rate of DMADDM.

The structures of DMADDM, PAMAM, and PD were tested by ^1^H NMR, which is shown in Fig. 1A. The assignable groups and chemical shifts are showed below: for DMADDM, *δ* (ppm): 3.46 (6H, s, C*H3*N), 5.52, 6.03 (4H, s, C*H2*CCH3C), 4.6 (2H, s, CH2C*H2*N), and 4.13 (2H, s, CH3C*H2*O), which was similar to the results reported before [49]. And for PAMAM, *δ* (ppm): 3.25–3.30 (–NH–CH_2_-CH_2_-N-, 12), 2.60–2.62 (–NH–CH_2_-CH_2_-N-, 12), 2.80–2.82 (-N–CH_2_–CH_2_-CO-, 28), 2.37–2.45 (-N–CH_2_–CH_2_-CO-, 28), 3.22–3.27 (–CH_2_–CH_2_-NH_2_, 16), and 2.72–2.77 (–CH_2_–CH_2_-NH_2_, 16), which was similar to the results showed before [36]. The peaks of PAMAM and DMADDM in the structure of PD were changed, indicating an interaction between the substances and that the complex PD was synthesized.

Zeta potential is an important parameter to describe the electrostatic interaction between particles in a dispersed system and the dispersed system under the influence of this electrical phenomenon; furthermore, it is very significant to study the physical stability of the drug dispersed system [50]. The results of Zeta potential evaluation are shown in Table 1. PAMAM had almost no surface charge, and that of DMADDM was 35.67 ± 1.23 mV, which is consistent with our previous findings [51]. The Zeta potential of PD was found to be 25.50 ± 0.92 mV. A Positive charge is one of the antibacterial mechanisms of DMADDM for the reason that a positively charged quaternary amine N+ has the ability to interact with the bacteria and damage the functioning of the membrane [49,52]. The results indicated that PD has antibacterial potential and the system of the complex is stable.

**Table 1:** Zeta potential in aqueous solution.

Agents	Concentration (mg/mL)	Zeta potential (mV)
PAMAM	1	−0.06 ± 0.03
DMADDM	1	35.67 ± 1.23
PD	1	25.50 ± 0.92

Particle size is also an important index for evaluating the biological performance of carrier systems [53]. The particle sizes are shown in Table 2. For PAMAM, the mean size was 201.90 ± 20.09 nm. The mean size of DMADDM was 2.36 ± 0.18 nm, whereas that of PD was 94.70 ± 3.02 nm. Compared to PAMAM, the mean size of PD decreased, the possible reason was the electrostatic attraction between PAMAM and DMADDM for their zeta potential (Table 1), and there should be hydrogen bonding, Van der Waals' force effect too [54,55]. Thus, the results showed that the average particle size of PAMAM changed after it was combined with DMADDM. Besides, PD was in the nanoscale range, which may be beneficial for its antibacterial effect. Accordingly, based on these results and the ^1^H NMR results, the compound PD was successfully synthesized.

**Table 2:** The particle size of DMADDM, PAMAM, and PD.

Agents	Concentration (mg/mL)	Size (nm)
PAMAM	1	201.90 ± 20.09
DMADDM	1	2.36 ± 0.18
PD	1	94.70 ± 3.02

Dendrimers are a kind of macromolecules which are three-dimensional spheres with branches emanating from the center [56]. The core of PAMAM forms a cavity [57]. Depending on the cavity and the branch architecture, plenty of drugs have been encapsulated within these cavities to delivered to the target area [36,58,59]. The amount of DMADDM encapsulated in PD was tested by ultraviolet absorbance. Fig. 1B showed a standard curve of DMADDM, and the concentration of unloaded DMADDM solution was 0.057 mg/mL, as the whole volume of the solution collected was 75 mL, and the solution was diluted 160 times before testing the concentration, so the loading efficiency and encapsulation efficiency of PD were measured to be 23.91% and 31.42%, respectively, indicating that DMADDM was loaded successfully (Fig. 1B). In a previous study, for testing the loaded drug of PAMAM, there were two methods. One was testing the weight of drugs released from the compound [36], and another way was testing the weight of unloaded drugs [58,59], and the loading efficiency of PAMAM was approximately between 0.5% and 20.59% and the encapsulation efficiency was between 7% and 92.5% [36,[58], [59], [60], [61], [62]]. The drug loading capacity of PD was high in our study. The release profiles of PD with the concentration of 7.8 mg/mL are shown in Fig. 1C. As per the results, during the first 80 h, there was a rapid release of DMADDM, and approximately 15% DMADDM was released from PAMAM. Subsequently, the rate of drug release gradually decreased. After three weeks, the DMADDM released from PAMAM was approximately 22%. In previous studies, for the release test of PAMAM, organic solvent was usually used to dissolve the compound, such as ethanol [36], methanol [58], and etc. The results above all showed that PD was a sustained-release compound, lasting for more than three weeks.

#### 3.1.2 Biosafety testing *in vitro*

Hemolysis is the release of hemoglobin through the rupture of red blood cells or through partially damaged membranes. *In vitro* hemolysis test is one of the most basic methods to detect blood compatibility and is also one of the important indices to detect the safety of biomaterials [63,64]. The detailed method could be seen in Supplementary materials, and the result of hemolysis test is shown in Supplementary Fig. 2. The Triton group acted as the positive control and showed complete hemolysis, whereas the phosphate buffered saline (PBS) group was the negative control and showed no hemolysis. According to the ISO 10993-4, when the drug is not applied through the vascular system, the hemolysis concentration below 9% (compared to Triton group, which showed 100% hemolysis) presents safe [65]. Therefore, PD at a concentration of 2 mg/mL or below will not cause detectable hemolysis and has good blood compatibility (Supplementary Fig. 2).

**Fig. 2:**
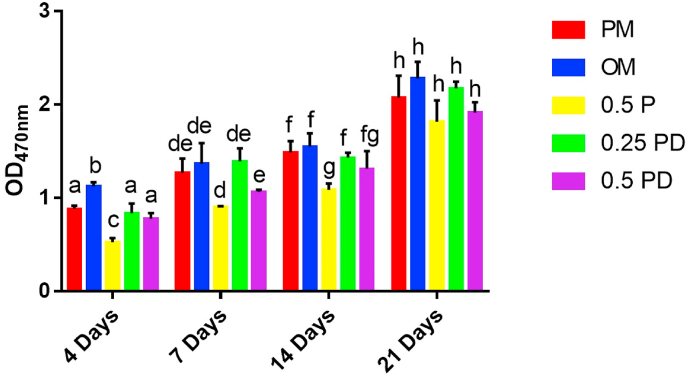
Proliferation of pre-osteoblasts. The different letters indicate the significant difference between the bars (a, b, c).

MC3T3-E1 is a pre-osteoblastic cell line, which has several biological characteristics such as alkaline phosphatase activity, type I collagen synthesis, and matrix calcification *in vitro*, and is often used as a cell model for bone metabolism research [66]. Proliferation of MC3T3-E1 cells is shown in Fig. 2. The results at 4 days showed that compared with the number of cells in the primary medium (PM, see the Materials and Methods section) group, the number of cells in the osteoinductive medium (OM) group increased (p < 0.01), whereas the cells in 0.5 mg/mL PAMAM group were significantly inhibited (p < 0.001). There was no significant difference between the 0.25 mg/mL PD group and 0.5 mg/mL PD group in this regard (p > 0.05). The results at 7 and 14 days showed that the proliferation of cells was partially inhibited in the 0.5 mg/mL PAMAM group (p < 0.05), whereas no statistically significant difference was observed in 0.25 mg/mL PD and 0.5 mg/mL PD groups (p > 0.05). The results at 21 days showed that the PM group was not significantly different from the other groups in terms of cellular proliferation (p > 0.05). The results indicated that 0.5 mg/mL PAMAM showed a partial inhibitory effect on the proliferation of MC3T3-E1 cells, whereas 0.25 mg/mL and 0.5 mg/mL PD showed no inhibitory effect.

#### 3.1.3 Biosafety testing *in vivo*

**Fig. 3:**
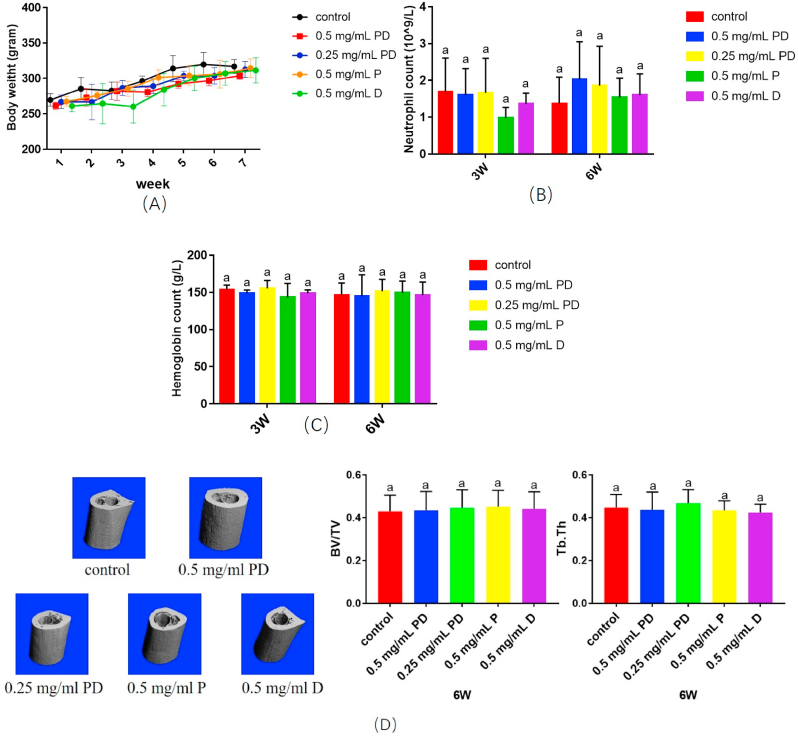
Cytotoxicity and biosafety testing of PD *in vivo*. (A) The body weight of rats changes over time; (B) Blood neutrophil count at three and six weeks; (C) Hemoglobin level at three and six weeks; (D)Micro-CT 3D images and quantitative analysis. The different letters indicate the significant difference between the bars (a, b, c).

The femur model is usually used for evaluating the dental implant materials, for the enclosed space facilitates the control of variable [[67], [68], [69]]. There were no exclusions cause no animal died in the study. The body weight of rats kept gradually increasing in all groups during the six-week, and no significant difference was observed between groups (p > 0.05, Fig. 3A). The result of neutrophil count and hemoglobin level showed no statistically different at three weeks and six weeks (p > 0.05, Fig. 3B and C). Hence, the inflammation of rats didn't show any difference. Furthermore, qualitative and quantitative of the bone around the Ti rod were showed in Fig. 3D. The result showed that BV/TV and Tb.Th were not significantly different between groups (p > 0.05). These results indicate that the novel compound PD significantly improved the biocompatibility of DMADDM, indicating that PD administration is safe *in vivo*.

Hematoxylin and eosin (HE) staining (Supplementary Fig. 3), and van Gieson's staining (Supplementary Fig. 4) were used to evaluate the morphological changes. HE staining showed no obvious difference between groups. As per the results on van Gieson's staining, no new bone formation seemed to be observed in the 0.5 mg/mL DMADDM group, and there was little new bone formation in the PBS group, whereas comparatively higher bone formation was observed in the PAMAM and PD groups. This indicated that PAMAM and PD did not impair new bone formation and even had the potential to promote osseointegration.

### 3.2 Anti-bacterial effect of PD + MAO *in vitro*

The traditional treatment of peri-implantitis includes repeated complete debridement, replacement of dental implants, and intravenous antibiotic administration, the efficacies of which are often limited [10]. Local antibiotic application is also a way to treat the infection; whereas, because of the overuse of antibiotics, antibiotic resistance increases the risk of a secondary infection in drug-resistant cases [11]. The antibacterial effect of PD results from DMADDM, which is less likely to cause microbial resistance compared with traditional agents such as chlorhexidine and antibiotics [29,70]. In some cases, although infection is not the initial problem, the presence of microorganism could initiate or accelerate the failure pathway [11]. Thus, antibacterial effects in the early stages of implantation are particular important [71].

**Fig. 4:**
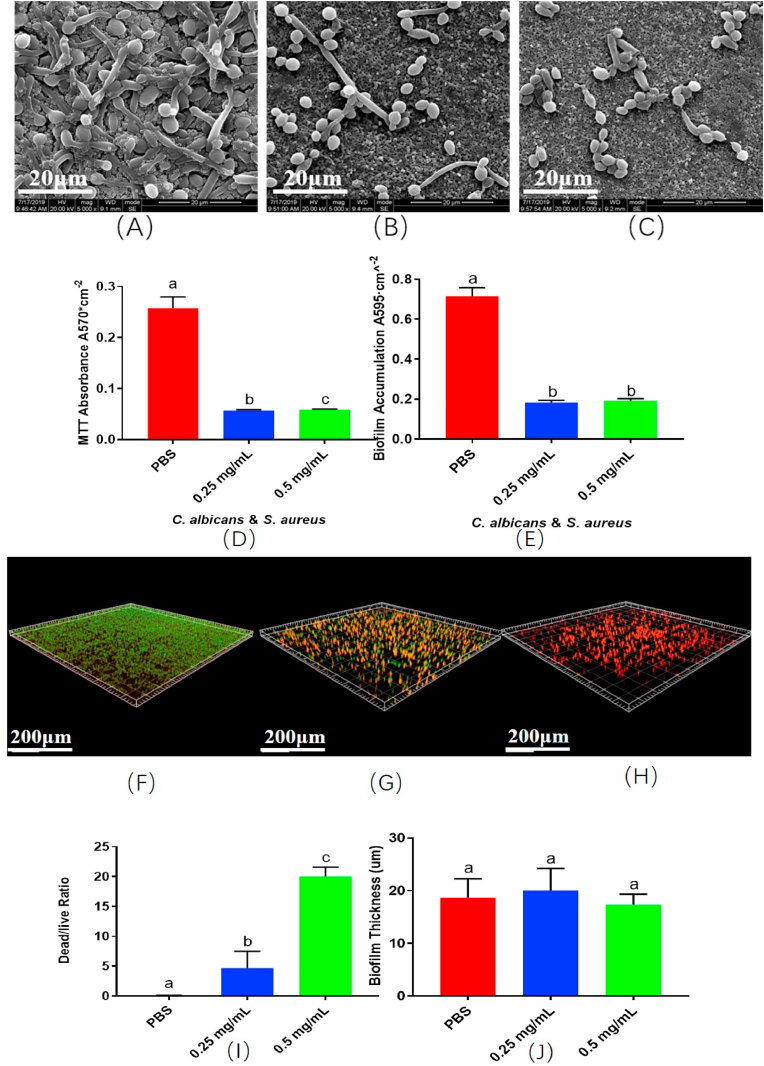
Anti-bacterial effect of PD combined with MAO implant *in vitro*. SEM images and confocal images of the biofilm (A)(F) PBS + MAO; (B)(G) 0.25 mg/mL PD + MAO; (C)(H) 0.5 mg/mL PD + MAO; (D) Metabolism analysis; (E) Biofilm accumulation; (I) Dead/live ratio; (J) Biofilm thickness. The different letters indicate the significant difference between the bars (a, b, c).

Once the biocompatibility of the PD was proved, we further investigated its antibacterial characteristics *in vitro*. As shown in Fig. 4A–C, with an increase in the mass fraction of PD, *S. aureus* as well as *C. albicans* concentrations decreased. As shown in Fig. 4D and E, the metabolism and biofilm accumulation in the 0.25 mg/mL group and 0.5 mg/mL group were significantly reduced (p＜0.05) compared with those in the PBS group; however, no significant difference was observed between the 0.25 mg/mL and 0.5 mg/mL groups (p > 0.05). This might be because the biofilm accumulation was too less to be calculated. The live/dead bacteria staining are showed in Fig. 4F–H. There were more dead cells in PD groups. Although the three groups showed no significant difference in biofilm thickness (Fig. 4J) (p > 0.05), the 0.5 mg/mL PD group had the largest dead/live ratio (Fig. 4I) (p < 0.05), and the ratio in the 0.25 mg/mL group was higher than that in the PBS group (Fig. 4I) (p < 0.05).

### 3.3 Anti-bacterial effect of PD + MAO *in vivo*

**Fig. 5:**
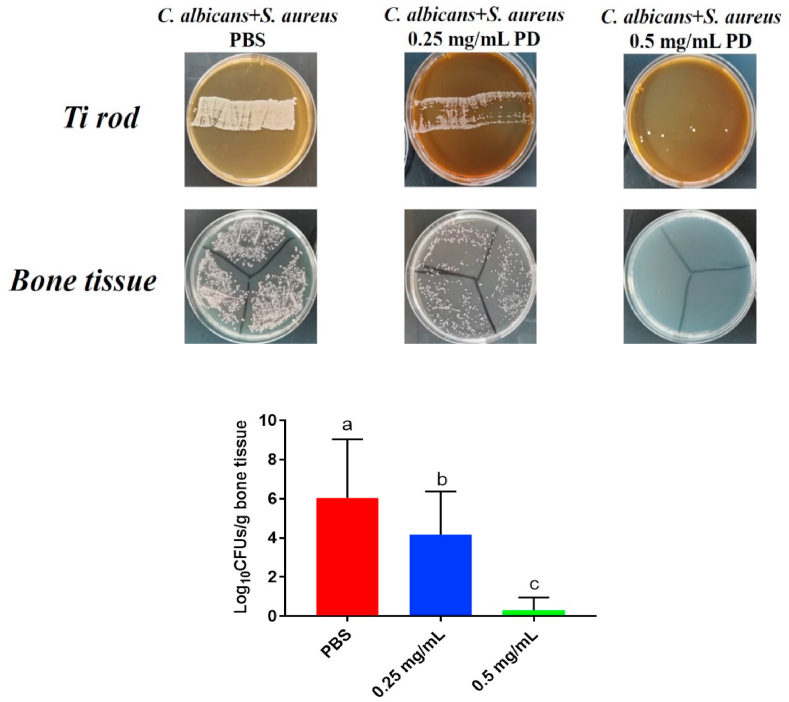
Anti-bacterial effect of PD combined with MAO implant *in vivo*. CFUs of bacteria on the Ti rod and around the bone tissue. The different letters indicate the significant difference between the bars (a, b, c).

To evaluate the infection prevention effects of PD + MAO, a rat peri-implantitis model using implants inoculated with *S. aureus* and *C. albicans* was used. After three weeks of implant insertion, the rats were sacrificed and the Ti rod and bone tissue were collected to perform the CFU assay. The bacteria around the Ti rod were tested for the adhesive biofilm while the bacteria in the bone tissue demonstrated the planktonic microorganism. With an increase in the mass fraction of PD, total microorganism levels decreased (p < 0.05). The total microorganism level around the Ti rod in the 0.25 mg/mL group and the 0.5 mg/mL group was reduced by 2-log and almost 5-log compared with that in the PBS group, respectively (Fig. 5).

The results indicated that PD + MAO could inhibit biofilms on the surface as well as decrease the planktonic microorganism in the bone tissue. By this way, the use of additional systemic antibiotic applications could be reduced. Based on the abovementioned results, PD + MAO have the ability to prevent infection during the very high-risk first stage.

### 3.4 Effect of PD + MAO on osteogenic differentiation *in vitro*

**Fig. 6:**
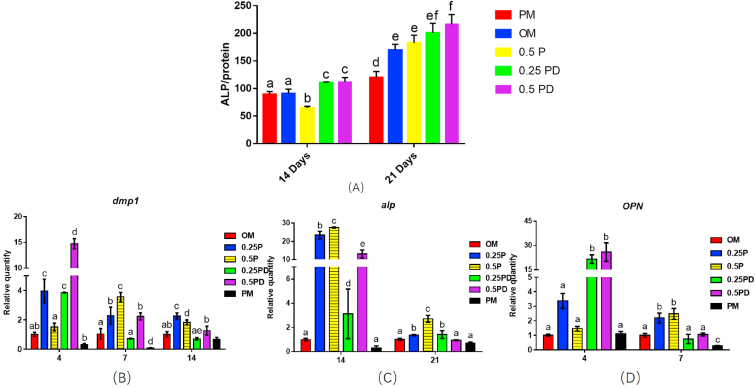
Effect of PD on osteogenic differentiation *in vitro*. (A) Alkaline phosphatase activity test; (B) Relative expression of *dmp1* gene; (C) Relative expression of *alp* gene; and (D) Relative expression of *OPN* gene. The different letters indicate the significant difference between the bars (a, b, c, d, e).

Osseointegration plays an important role in implants stabilization. We first investigated the osseointegration properties of PD *in vitro*. The ALP activity test is shown in Fig. 6A. The results at 14 days showed that the ALP activity in the 0.5 mg/mL PAMAM group decreased compared with that in the PM group (p < 0.01), whereas the ALP activity in the 0.25 and 0.5 mg/mL PD groups were higher (p < 0.01) than that in the PM group. At 21 days, the ALP activities in the OM group and the 0.5 mg/mL PAMAM group were higher than those in the PM group (p < 0.01); furthermore, the 0.25 mg/mL and 0.5 mg/mL PD groups showed significantly increase ALP activity at 21 days (p < 0.001), indicating a certain concentration dependence.

The relative expression of the *alp* gene is shown in Fig. 6C. At 14 days, the gene expression levels in 0.25 mg/mL PAMAM (p < 0.001), 0.5 mg/mL PAMAM (p < 0.001), 0.25 mg/mL PD (p < 0.05), and 0.5 mg/mL PD groups (p < 0.001) were significantly increased compared with the level in the OM group. At 21 days, the gene expression level in the 0.25 mg/mL PAMAM, 0.5 mg/mL PAMAM and 0.25 mg/mL PD groups was increased (p < 0.05). The result was consistent with the ALP activity test results (Fig. 6A). *OPN* gene expression levels are shown in Fig. 6D. At 4 days, the gene expression was significantly increased in the 0.25 mg/mL PD group and 0.5 mg/mL PD group compared with that in the PM group (p < 0.001). At 7 days, the gene expression levels in the 0.25 mg/mL PAMAM group and 0.5 mg/mL PAMAM group were increased (p < 0.05). Expression levels of *dmp1* in different groups are shown in Fig. 6B. At 4 days, compared with OM group, the expression levels were significantly increased in the 0.25 mg/mL PAMAM, 0.25 mg/mL PD, and 0.5 mg/mL PD groups (p < 0.001). At 7 days, the gene expression levels were significantly increased in the 0.25 mg/mL PAMAM, 0.5 mg/mL PAMAM (p < 0.001), and 0.5 mg/mL PD groups (p < 0.01) compared with OM group. Furthermore, at 14 days, the gene expression levels in the 0.25 mg/mL PAMAM (p < 0.001) and 0.5 mg/mL PAMAM group (p < 0.05) were significantly increased, whereas those in the PD groups did not significantly increase.

ALP, which is associated with the formation of hydroxyapatite crystals in osteoblasts, is a representative protein product of the osteoblast activity and is a good indicator of osteoblast differentiation. ALP activity is the most widely recognized biochemical marker for detecting osteoblastic activity [72,73]. Osteopontin (OPN) is one of the most abundant non-collagen proteins in bone matrix [74]. *In vitro* studies have found that *OPN* mRNA levels in osteoblasts increase when the bone matrix starts to mineralize; accordingly, *OPN* is also an osteogenesis-related gene [75]. *Dentin matrix protein 1* (*dmp1*) was found in 1993, and was thought to be a dentin-specific protein at first [76]. However, later studies showed that the mineralization occurs earlier and the mineralized nodules are larger in MC3T3-E1 cells when the gene *dmp1* is overexpressed [77]. According to the abovementioned results, PAMAM increased the expression level of *dmp1* gene in the early stage (at 4 days and 7 days) and increased the expression level of *dmp1* and *alp* gene in the middle stage (at 14 days). PD increased the expression levels of *dmp1* and *OPN* in the early stage and increased the levels of *alp* in the middle stage. This demonstrates that PD has the potential to promote osteogenic differentiation of MC3T3-E1 cells.

### 3.5 Effect of PD + MAO on osteogenic differentiation *in vivo*

**Fig. 7:**
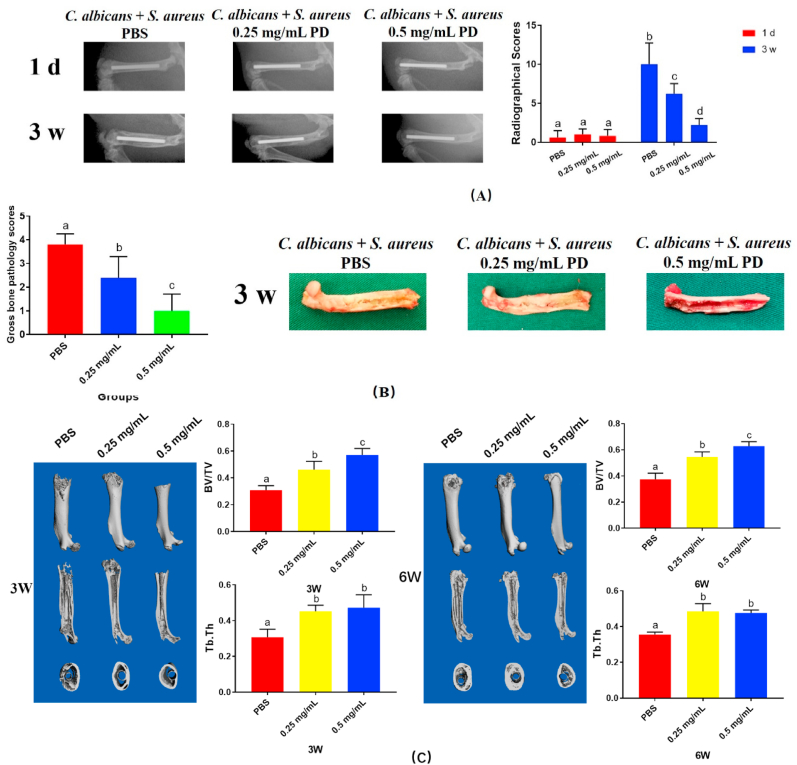
Effect of PD + MAO on osteogenic differentiation *in vivo*. (A) The X-ray images and scores; (B) Gross images and scores; (C) Micro-CT images and BV/TV and Tb.Th. The different letters indicate the significant difference between the bars (a, b, c, d).

X-ray evaluation was showed in Fig. 7A. The result demonstrated that the rats in the PBS group represented periosteal reactions and osteolysis, and in the PD groups, there were less bone infection. On the day of implantation, radiographic score evaluation indicated that there was no differences between groups, whereas significant differences were showed among these groups after 3 weeks (p < 0.05, Fig. 7A).

Furthermore, severe pus formation was observed in the PBS group. Whereas, in the PD groups, the presence of less pus formation indicated that the clinical signs of pyogenic infections were greatly reduced. With an increase in the mass fraction of PD, the gross score significantly decreased (p < 0.05, Fig. 7B).

We conducted qualitative and quantitative analysis of the bone tissue around the Ti rod subsequently. The analyses conducted at 3 weeks and 6 weeks post-surgery are showed in Fig. 7C. In the PD groups, there was newly formed bone around the implant. The PBS group showed massive destruction of bone tissue. The results were consistent with the radiographic results shown in Fig. 6A. In addition, the BV/TV and Tb.Th in the PD groups were significantly higher than PBS group (p < 0.05), showing that bone loss was less in the PD groups.

The morphological changes were observed by HE (Supplementary Fig. 5), and van Gieson's staining (Supplementary Fig. 6). There were apparent medullary sequestrum formation, bone loss and fibrosis observed in the PBS group. By comparison, the implants in the 0.25 mg/mL PD and 0.5 mg/mL PD groups indicated no evident bone loss. As shown by van Gieson's staining, the hard tissue stained in red; very little new bone was observed around the implant in the PBS group in 6 weeks, whereas more bone was observed around the implant in the 0.25 mg/mL PD group; furthermore, the 0.5 mg/mL PD group showed more bone formation compared with the 0.25 mg/mL PD group, which indicated that PD could promote bone formation.

After three weeks, the rats in the PD groups showed less inflammation and bone destruction. Furthermore, compared with the rats in the PBS group, the rats in the PD groups showed reduced pus formation, lower radiographic and gross pathology scores. According to the data above, the use of MAO + PD is a successfully application for treating peri-implantitis. In the high-risk early stage, PD showed strong antibacterial and antifungal effects as well as low drug resistance, which was largely because of DMADDM. Besides, the cytotoxicity of PD was decreased because of the loading and slow releasing of PAMAM, therefore, allowing the new bone formation and osseointegration to happen. Moreover, in this study, PAMAM itself had the potential to promote osseointegration, which may be because of its ability to act as a scaffold for hard tissue regeneration. In the second stage, MAO has the potential to promote osteogenesis while maintaining a certain antibacterial effect; this was confirmed in the *in vivo* study. Hence, this is a double benefits material with great promise to prevent peri-implantitis during the two stages of implantation. Nonetheless, the fabrication process of PD still needs to be optimized. Because the PD was applied like a local drug, coating the PD on titanium are beneficial to the osteointegration due to the PAMAM retained on the implant surface after the DMADDM released. What's more, the two-staged double benefits dental implant need to be tested in larger animals.

## 4 Conclusion

This study demonstrated a PD + MAO dental implant with two-stage double benefits. In the high-risk first stage, PD enables to decrease the adherence and development of microorganisms by releasing DMADDM. Then, PD as well as MAO can promote osteogenesis and osseointegration. Furthermore, the PD + MAO strategy was proved to have great anti-infection and osteoconductive characteristics in a peri-implantitis rat model *in vivo*. Hence, PD + MAO is a kind of two-stage double benefits strategy of infection prevention and osteogenesis promotion. The modified dental implant may have a potential application in future clinical practice.

## CRediT authorship contribution statement

**Xiaoyu Huang:** Conceptualization, Methodology, Investigation, Writing – original draft. **Yang Ge:** Conceptualization, Methodology, Investigation, Writing – original draft. **Bina Yang:** Conceptualization, Methodology, Investigation, Writing – original draft. **Qi Han:** Investigation. **Wen Zhou:** Investigation. **Jingou Liang:** Investigation. **Mingyun Li:** Writing – review & editing. **Xian Peng:** Writing – review & editing. **Biao Ren:** Writing – review & editing. **Bangcheng Yang:** Writing – review & editing. **Michael D. Weir:** Investigation. **Qiang Guo:** Writing – review & editing. **Haohao Wang:** Investigation. **Xinxuan Zhou:** Investigation. **Xugang Lu:** Investigation. **Thomas W. Oates:** Writing – review & editing. **Hockin H.K. Xu:** Conceptualization, Writing – review & editing, Project administration. **Dongmei Deng:** Conceptualization, Writing – review & editing, Project administration. **Xuedong Zhou:** Conceptualization, Writing – review & editing, Project administration, Funding acquisition. **Lei Cheng:** Conceptualization, Writing – review & editing, Project administration, Funding acquisition.

## Declaration of competing interest

The authors declare no conflict of interest.

## References

[1] Schwarz F., Derks J., Monje A., Wang H.L. (2018). Peri-implantitis. J. Periodontol..

[2] Mameno T., Wada M., Onodera Y., Fujita D., Sato H., Ikebe K. (2018). Longitudinal study on risk indicators for peri-implantitis using survival-time analysis. J. Prosthodont Res..

[3] Schwendicke F., Tu Y.K., Stolpe M. (2015). Preventing and treating peri-implantitis: a cost-effectiveness analysis. J. Periodontol..

[4] Cataldo M.A., Petrosillo N., Cipriani M., Cauda R., Tacconelli E. (2010). Prosthetic joint infection: recent developments in diagnosis and management. J. Infect..

[5] Ercan B., Kummer K.M., Tarquinio K.M., Webster T.J. (2011). Decreased Staphylococcus aureus biofilm growth on anodized nanotubular titanium and the effect of electrical stimulation. Acta Biomater..

[6] Zilberman M., Elsner J.J. (2008). Antibiotic-eluting medical devices for various applications. J. Contr. Release.

[7] Bouza E., Burillo A., Munoz P., Guinea J., Marin M., Rodriguez-Creixems M. (2013). Mixed bloodstream infections involving bacteria and Candida spp. J. Antimicrob. Chemother..

[8] Harriott M.M., Noverr M.C. (2009). Candida albicans and Staphylococcus aureus form polymicrobial biofilms: effects on antimicrobial resistance. Antimicrob. Agents Chemother..

[9] Adam B., Baillie G.S., Douglas L.J. (2002). Mixed species biofilms of Candida albicans and Staphylococcus epidermidis. J. Med. Microbiol..

[10] Cook G.E., Markel D.C., Ren W., Webb L.X., McKee M.D., Schemitsch E.H. (2015). Infection in Orthopaedics. J Orthop Trauma.

[11] Raphel J., Holodniy M., Goodman S.B., Heilshorn S.C. (2016). Multifunctional coatings to simultaneously promote osseointegration and prevent infection of orthopaedic implants. Biomaterials.

[12] Romano C.L., Scarponi S., Gallazzi E., Romano D., Drago L. (2015). Antibacterial coating of implants in orthopaedics and trauma: a classification proposal in an evolving panorama. J. Orthop. Surg. Res..

[13] Esteban J., Molina-Manso D., Spiliopoulou I., Cordero-Ampuero J., Fernandez-Roblas R., Foka A., Gomez-Barrena E. (2010). Biofilm development by clinical isolates of Staphylococcus spp. from retrieved orthopedic prostheses. Acta Orthop..

[14] Pan C., Zhou Z., Yu X. (2018). Coatings as the useful drug delivery system for the prevention of implant-related infections. J. Orthop. Surg. Res..

[15] Zimmerli W. (2014). Clinical presentation and treatment of orthopaedic implant-associated infection. J. Intern. Med..

[16] Arciola C.R., Campoccia D., Montanaro L. (2018). Implant infections: adhesion, biofilm formation and immune evasion. Nat. Rev. Microbiol..

[17] Hickok N.J., Shapiro I.M. (2012). Immobilized antibiotics to prevent orthopaedic implant infections. Adv. Drug Deliv. Rev..

[18] Zimmerli W., Ochsner P.E. (2003). Management of infection associated with prosthetic joints. Infection.

[19] Zhou W., Peng X., Ma Y., Hu Y., Wu Y., Lan F., Weir M.D., Li M., Ren B., Oates T.W., Xu H.H.K., Zhou X., Cheng L. (2019). Two-staged time-dependent materials for the prevention of implant-related infections. Acta Biomater..

[20] Campoccia D., Montanaro L., Arciola C.R. (2013). A review of the biomaterials technologies for infection-resistant surfaces. Biomaterials.

[21] Alt V. (2017). Antimicrobial coated implants in trauma and orthopaedics-A clinical review and risk-benefit analysis. Injury.

[22] Li X., Qi M., Sun X., Weir M.D., Tay F.R., Oates T.W., Dong B., Zhou Y., Wang L., Xu H.H.K. (2019). Surface treatments on titanium implants via nanostructured ceria for antibacterial and anti-inflammatory capabilities. Acta Biomater..

[23] Li B., Ge Y., Wu Y., Chen J., Xu H.H.K., Yang M., Li M., Ren B., Feng M., Weir M.D., Peng X., Cheng L., Zhou X. (2017). Anti-bacteria and microecosystem-regulating effects of dental implant coated with dimethylaminododecyl methacrylate. Molecules.

[25] Mas-Moruno C., Su B., Dalby M.J. (2019). Multifunctional coatings and nanotopographies: toward cell instructive and antibacterial implants. Adv. Healthc. Mater..

[26] Lu X., Xiong S., Chen Y., Zhao F., Hu Y., Guo Y., Wu B., Huang P., Yang B. (2020). Effects of statherin on the biological properties of titanium metals subjected to different surface modification. Colloids Surf. B Biointerfaces.

[27] Huang X., Zhou W., Zhou X.D., Hu Y., Xiang P., Li B., Yang B., Peng X., Li M., Cheng L. (2019). Effect of novel micro-arc oxidation implant Material on Preventing Peri-Implantitis. Coatings.

[28] Cheng L., Zhang K., Zhang N., Melo M.A.S., Weir M.D., Zhou X.D., Bai Y.X., Reynolds M.A., Xu H.H.K. (2017). Developing a new generation of antimicrobial and bioactive dental resins. J. Dent. Res..

[29] Wang S., Wang H., Ren B., Li H., Weir M.D., Zhou X., Oates T.W., Cheng L., Xu H.H.K. (2017). Do quaternary ammonium monomers induce drug resistance in cariogenic, endodontic and periodontal bacterial species?. Dent. Mater..

[30] Cheng L., Weir M.D., Zhang K., Arola D.D., Zhou X., Xu H.H. (2013). Dental primer and adhesive containing a new antibacterial quaternary ammonium monomer dimethylaminododecyl methacrylate. J. Dent..

[31] Wang S., Zhang K., Zhou X., Xu N., Xu H.H., Weir M.D., Ge Y., Wang S., Li M., Li Y., Xu X., Cheng L. (2014). Antibacterial effect of dental adhesive containing dimethylaminododecyl methacrylate on the development of Streptococcus mutans biofilm. Int. J. Mol. Sci..

[32] Zhou Y., Yang J., Lin Z., Li J., Liang K., Yuan H., Li S., Li J. (2014). Triclosan-loaded poly(amido amine) dendrimer for simultaneous treatment and remineralization of human dentine. Colloids Surf. B Biointerfaces.

[33] Liang J., Peng X., Zhou X., Zou J., Cheng L. (2020). Emerging applications of drug delivery systems in oral infectious diseases prevention and treatment. Molecules.

[34] Staehlke S., Lehnfeld J., Schneider A., Nebe J.B., Muller R. (2019). Terminal chemical functions of polyamidoamine dendrimer surfaces and its impact on bone cell growth. Mater. Sci. Eng. C Mater. Biol. Appl..

[35] Yue C., Yang B. (2014). Bioactive titanium surfaces with the effect of inhibiting biofilm formation. J. Bionic Eng..

[36] Zhu B., Li X., Xu X., Li J., Ding C., Zhao C., Li J. (2018). One-step phosphorylated poly(amide-amine) dendrimer loaded with apigenin for simultaneous remineralization and antibacterial of dentine. Colloids Surf. B Biointerfaces.

[37] Papadimitriou S., Bikiaris D. (2009). Novel self-assembled core-shell nanoparticles based on crystalline amorphous moieties of aliphatic copolyesters for efficient controlled drug release. J. Contr. Release.

[38] Tan Y., Leonhard M., Moser D., Ma S., Schneider-Stickler B. (2019). Antibiofilm efficacy of curcumin in combination with 2-aminobenzimidazole against single- and mixed-species biofilms of Candida albicans and Staphylococcus aureus. Colloids Surf. B Biointerfaces.

[39] Percie du Sert N., Hurst V., Ahluwalia A., Alam S., Avey M.T., Baker M., Browne W.J., Clark A., Cuthill I.C., Dirnagl U., Emerson M., Garner P., Holgate S.T., Howells D.W., Karp N.A., Lazic S.E., Lidster K., MacCallum C.J., Macleod M., Pearl E.J., Petersen O.H., Rawle F., Reynolds P., Rooney K., Sena E.S., Silberberg S.D., Steckler T., Wurbel H. (2020). The ARRIVE guidelines 2.0: updated guidelines for reporting animal research. PLoS Biol..

[40] Nie B., Ao H., Long T., Zhou J., Tang T., Yue B. (2017). Immobilizing bacitracin on titanium for prophylaxis of infections and for improving osteoinductivity: an in vivo study. Colloids Surf. B Biointerfaces.

[41] Yang Y., Ao H., Wang Y., Lin W., Yang S., Zhang S., Yu Z., Tang T. (2016). Cytocompatibility with osteogenic cells and enhanced in vivo anti-infection potential of quaternized chitosan-loaded titania nanotubes. Bone Res..

[42] Campoccia D., Montanaro L., Arciola C.R. (2006). The significance of infection related to orthopedic devices and issues of antibiotic resistance. Biomaterials.

[43] Lovati A.B., Bottagisio M., de Vecchi E., Gallazzi E., Drago L. (2017). Animal models of implant-related low-grade infections. A twenty-year review. Adv. Exp. Med. Biol..

[44] Arciola C.R., Campoccia D., Ehrlich G.D., Montanaro L. (2015). Biofilm-based implant infections in orthopaedics. Adv. Exp. Med. Biol..

[45] Schlecht L.M., Peters B.M., Krom B.P., Freiberg J.A., Hansch G.M., Filler S.G., Jabra-Rizk M.A., Shirtliff M.E. (2015). Systemic Staphylococcus aureus infection mediated by Candida albicans hyphal invasion of mucosal tissue. Microbiology.

[46] Kucharikova S., Gerits E., De Brucker K., Braem A., Ceh K., Majdic G., Spanic T., Pogorevc E., Verstraeten N., Tournu H., Delattin N., Impellizzeri F., Erdtmann M., Krona A., Lovenklev M., Knezevic M., Frohlich M., Vleugels J., Fauvart M., de Silva W.J., Vandamme K., Garcia-Forgas J., Cammue B.P., Michiels J., Van Dijck P., Thevissen K. (2016). Covalent immobilization of antimicrobial agents on titanium prevents Staphylococcus aureus and Candida albicans colonization and biofilm formation. J. Antimicrob. Chemother..

[47] Smojver I., Vuletić M., Gerbl D., Budimir A., Sušić M., Gabrić D. (2021). Evaluation of antimicrobial efficacy and permeability of various sealing materials at the implant–abutment interface—a pilot in vitro study. Materials.

[48] Zhou W., Peng X., Zhou X., Li M., Ren B., Cheng L. (2019). Influence of bio-aging on corrosion behavior of different implant materials. Clin. Implant Dent. Relat. Res..

[49] Liang J., Li M., Ren B., Wu T., Xu H.H.K., Liu Y., Peng X., Yang G., Weir M.D., Zhang S., Cheng L., Zhou X. (2018). The anti-caries effects of dental adhesive resin influenced by the position of functional groups in quaternary ammonium monomers. Dent. Mater..

[50] Al Mahrouqi D., Vinogradov J., Jackson M.D. (2017). Zeta potential of artificial and natural calcite in aqueous solution. Adv. Colloid Interface Sci..

[51] Han Q., Li B., Zhou X., Ge Y., Wang S., Li M., Ren B., Wang H., Zhang K., Xu H.H.K., Peng X., Feng M., Weir M.D., Chen Y., Cheng L. (2017). Anti-Caries effects of dental adhesives containing quaternary ammonium methacrylates with different chain lengths. Materials.

[52] Yu J., Huang X., Zhou X., Han Q., Zhou W., Liang J., Xu H.H.K., Ren B., Peng X., Weir M.D., Li M., Cheng L. (2020). Anti-caries effect of resin infiltrant modified by quaternary ammonium monomers. J. Dent..

[53] Bhattacharjee S. (2016). DLS and zeta potential - what they are and what they are not?. J. Contr. Release.

[54] Marie P.G., Pettit W., Ferruti Paolo, Richardson Simon C.W. (2011). Poly(amidoamine) polymers: soluble linear amphiphilic drug-delivery systems for genes, proteins and oligonucleotides. Ther. Deliv..

[55] Li J., Liang H., Liu J., Wang Z. (2018). Poly (amidoamine) (PAMAM) dendrimer mediated delivery of drug and pDNA/siRNA for cancer therapy. Int. J. Pharm..

[56] Gillies E., Frechet J. (2005). Dendrimers and dendritic polymers in drug delivery. Drug Discov. Today.

[57] Kurtoglu Y.E., Mishra M.K., Kannan S., Kannan R.M. (2010). Drug release characteristics of PAMAM dendrimer-drug conjugates with different linkers. Int. J. Pharm..

[58] Pishavar E., Ramezani M., Hashemi M. (2019). Co-delivery of doxorubicin and TRAIL plasmid by modified PAMAM dendrimer in colon cancer cells, in vitro and in vivo evaluation. Drug Dev. Ind. Pharm..

[59] Thanh V.M., Nguyen T.H., Tran T.V., Ngoc U.P., Ho M.N., Nguyen T.T., Chau Y.N.T., Le V.T., Tran N.Q., Nguyen C.K., Nguyen D.H. (2018). Low systemic toxicity nanocarriers fabricated from heparin-mPEG and PAMAM dendrimers for controlled drug release. Mater. Sci. Eng. C Mater. Biol. Appl..

[60] Mekonnen T.W., Andrgie A.T., Darge H.F., Birhan Y.S., Hanurry E.Y., Chou H.Y., Lai J.Y., Tsai H.C., Yang J.M., Chang Y.H. (2020). Bioinspired composite, pH-responsive sodium deoxycholate hydrogel and generation 4.5 poly(amidoamine) dendrimer improves cancer treatment efficacy via doxorubicin and resveratrol Co-delivery. Pharmaceutics.

[61] Luong D., Kesharwani P., Killinger B.A., Moszczynska A., Sarkar F.H., Padhye S., Rishi A.K., Iyer A.K. (2016). Solubility enhancement and targeted delivery of a potent anticancer flavonoid analogue to cancer cells using ligand decorated dendrimer nano-architectures. J. Colloid Interface Sci..

[62] Dichwalkar T., Patel S., Bapat S., Pancholi P., Jasani N., Desai B., Yellepeddi V.K., Sehdev V. (2017). Omega-3 fatty acid grafted PAMAM-paclitaxel conjugate exhibits enhanced anticancer activity in upper gastrointestinal cancer cells. Macromol. Biosci..

[63] Fan X.L., Hu M., Qin Z.H., Wang J., Chen X.C., Lei W.X., Ye W.Y., Jin Q., Ren K.F., Ji J. (2018). Bactericidal and hemocompatible coating via the mixed-charged copolymer. ACS Appl. Mater. Interfaces.

[64] Evans B.C., Nelson C.E., Yu S.S., Beavers K.R., Kim A.J., Li H., Nelson H.M., Giorgio T.D., Duvall C.L. (2013). Ex vivo red blood cell hemolysis assay for the evaluation of pH-responsive endosomolytic agents for cytosolic delivery of biomacromolecular drugs. J. Vis. Exp..

[65] Seyfert U.T., Biehl V. (2002). In vitro hemocompatibility testing of biomaterials according to the ISO 10993-4. Biomol. Eng..

[66] Sudo H., Kodama H.A. (1983). In vitro differentiation and calcification in a new clonal osteogenic cell line derived from newborn mouse calvaria. JCB (J. Cell Biol.).

[67] Yang W.E., Huang H.H. (2021). TiO2 nanonetwork on rough Ti enhanced osteogenesis in vitro and in vivo. J. Dent. Res..

[68] Shu T., Zhang Y., Sun G., Pan Y., He G., Cheng Y., Li A., Pei D. (2020). Enhanced osseointegration by the hierarchical micro-nano topography on selective laser melting Ti-6Al-4V dental implants. Front Bioeng. Biotechnol..

[69] Liu W., Zhou L., Xue H., Li H., Yuan Q. (2020). Growth differentiation factor 11 impairs titanium implant healing in the femur and leads to mandibular bone loss. J. Periodontol..

[70] Jiang Y.-L., Qiu W., Zhou X.-D., Li H., Lu J.-Z., Xu H.H.K., Peng X., Li M.-Y., Feng M.-Y., Cheng L., Ren B. (2017). Quaternary ammonium-induced multidrug tolerant Streptococcus mutans persisters elevate cariogenic virulence in vitro. Int. J. Oral Sci..

[71] Chouirfa H., Bouloussa H., Migonney V., Falentin-Daudre C. (2019). Review of titanium surface modification techniques and coatings for antibacterial applications. Acta Biomater..

[72] Vijayan V., Gupta S., Gupta S. (2017). Bone morphogenetic protein-5, a key molecule that mediates differentiation in MC3T3E1 osteoblast cell line. Biofactors.

[73] An J., Yang H., Zhang Q., Liu C., Zhao J., Zhang L., Chen B. (2016). Natural products for treatment of osteoporosis: the effects and mechanisms on promoting osteoblast-mediated bone formation. Life Sci..

[74] Martin V., Ribeiro I.A., Alves M.M., Goncalves L., Claudio R.A., Grenho L., Fernandes M.H., Gomes P., Santos C.F., Bettencourt A.F. (2019). Engineering a multifunctional 3D-printed PLA-collagen-minocycline-nanoHydroxyapatite scaffold with combined antimicrobial and osteogenic effects for bone regeneration. Mater. Sci. Eng. C Mater. Biol. Appl..

[75] Owen T.A., Aronow M. (1990). Progressive development of the rat osteoblast phenotype in vitro: reciprocal relationships in expression of genes associated with osteoblast proliferation and differentiation during formation. J. Cell. Physiol..

[76] George A., Sabsay B., A S.P. (1993). Characterization of a novel dentin matrix acidic phosphoprote. Implications for induction of biomineralization. J. Biol. Chem..

[77] Qin C., D'Souza R., Feng J.Q. (2007). Dentin matrix protein 1 (DMP1): new and important roles for biomineralization and phosphate homeostasis. J. Dent. Res..

