# Supplementary material for: Novel dental implant modifications with two-staged double benefits for preventing infection and promoting osseointegration in vivo and in vitro
Source: Bioact Mater. 2021 May 15;6(12):4568–79. doi: 10.1016/j.bioactmat.2021.04.041 (PMC8141509; doi:10.1016/j.bioactmat.2021.04.041)
Supplement: Multimedia component 1 [file mmc1.docx]

**Supplementary Material**

**Novel dental implant modifications with two-staged double benefits for preventing infection and promoting osseointegration *in vivo* and *in vitro***

**Methods:**

***Multi-species biofilm formation***

*Staphylococcus aureus* (ATCC 25923) and *Candida albicans* (SC-5314）were used in this study for monomicrobial and polymicrobial biofilm formations. Yeast Peptone Dextrose (YPD, Sigma-Aldrich, Austria) medium and brain-heart infusion broth (BHI; Difco; Sparks, MD, USA) were used for culturing *C. albicans* and *S. aureus*, respectively. RPMI1640 medium (GlutaMAX™ Supplement, Thermo Fisher Scientific, USA) was chosen for supporting the growth of the biofilms. The bacterial concentration was adjusted to 1 × 10^6^ CFU/mL and incubated in anerobic environment for 24 h.

***Crystal Violet Assay and MTT Assay***

The crystal violet assay was performed to determine biomass accumulation. The PBS-rinsed biofilms were placed into a 24-well plate. Each biofilm was submerged in 1 mL 100% methyl alcohol for 15 min for fixation. Subsequently, biofilms on the disks were rinsed with PBS and transferred to a new 24-well plate and submerged in 1 mL 0.1% crystal violet solution for 5 min. To remove the residual dye, the biofilms were again washed with PBS. Following this, the disks were transferred to another 24-well plate. Then 2 mL of 95% ethanol solution was added to each well and the plate was shaken horizontally at 80 rpm for 45 min at room temperature. Subsequently, 100 µL of ethanol solution from each well was diluted with 95% ethanol solution to a total volume of 200 µL and transferred to a 96-well plate. A microplate reader was used to measure the absorbance of the solution at the optical density (OD) of 595 nm [1].

The 3-(4,5-Dimethyl-thiazol-2-yl)-2,5-diphenyltetrazolium bromide (MTT) assay was performed to measure the metabolic activity. The PBS-rinsed biofilm on the disk was placed into a 24-well plate and 1 mL MTT dye (0.5 mg/mL MTT in PBS) was added to each well. This biofilm plate was then cultured for 1 h (37℃ anaerobically). To dissolve the formazan crystals, the disks were fitted into a new 24-well plate filled with 2 mL dimethyl sulfoxide (DMSO) and shaken horizontally at 80 rpm for 20 min in dark. Finally, 200 µL of the DMSO solution containing the formazan crystals retained by the biofilms was pipetted into a 96-well plate to measure the absorbance at an OD of 540 nm using a microplate reader [1].

***Relative quantification of differentially expressed genes by qPCR***

The cells were harvested by centrifugation at 6000 r·min^-1^at 4°C for 5 min. The pellets were flash frozen in liquid nitrogen and stored at −80 °C until RNA preparation. RNA isolation was performed according to the instructions mentioned in the GeneJET RNA purification Kit (Thermo scientific). Subsequently, cDNA was prepared with 1 µg RNA using the One Step RNA PCR kit (Takara Inc.) according to the manufacturer’s instructions. The purity and concentration of DNA were detected using the NanoDrop 2000 spectrophotometer (Thermo Scientific, Waltham, MA, USA). RT-PCR was then performed using the SYBR® PremixEx TaqTM kit (Takara Inc.) following a two-step strategy: (1) 94°C for 30 s and (2) 40 PCR cycles (94°C for 30 s, a gene-specific annealing temperature for 30 s). All primer sequences are listed in Table S2. Real-time PCR of triplicate samples were performed using CFX 96 TouchTM (Bio-Rad, Hercules, CA, USA). The gene expression level relative to the calibrator was expressed as 2^−ΔΔCT^.

***Live/Dead Bacteria Staining***

For live/dead staining, the disks with biofilms were stained using the BacLight live/dead bacterial viability kit (Molecular Probes, Eugene, OR, USA) as per the manufacturer’s instructions. Live bacterial cells were stained to produce green fluorescence with SYTO 9, whereas cells with compromised membranes were stained red with propidium iodide. Three disks were examined using confocal laser scanning microscopy (Leica, Wetzlar, Germany). Three disks of each group were examined and the tests were repeated three times. The ratios of dead to live bacterial cells were calculated and partly quantified analysis of EPS production and bacteria cells were performed with Image-Pro Plus 6.0 (Media Cybernetics, Bethesda, MD, USA) and Matrix Laboratory (Mathworks, MA, USA) by calculating the value of relative fluorescence [1, 2].

***SEM Observation***

For scanning electron microscopy (SEM) examination, the PBS-rinsed biofilms on the disks were immersed in 1% glutaraldehyde for 1 h at 4℃. Then, the disks were washed twice in sterile water (immersion time per wash, 10 min) and dehydrated in a series of graded ethanol solutions (50%, 60%, 70%, 80%, 90%, and 100%; immersion time per series, 10 min), followed by sputter-coating with gold. Finally, SEM (Quanta 200, FEI, Hillsboro, OR, USA) was performed to examine the biofilms [1, 3, 4].

***Cytotoxicity***

Mouse preosteoblasts (MC3T3-E1; Sigma) were inoculated in a primary medium (PM), which was an α-MEM medium containing 10% fetal bovine serum. The osteoinductive medium (OM) included the PM with 50 g/mL vitamin C ascorbic acid and 10mM glycerophosphate. Cells were seeded in 24-well plate at a density of 1 × 10^5^ cells/mL. All groups were incubated in an incubator at a constant temperature of 37℃ under 5% CO_2_ for 24 h. After the cells covered 80% of the plate, the medium was refreshed with PM, OM, OM + 0.25 mg/ml PD, OM + 0.5 mg/ml PD, or OM + 0.5 mg/mL PAMAM. After 24 h, the medium was replaced with OM or PM every two days [5].

At days 4, 7, 14, and 21, the original medium in the 24-well plate was discarded and the cells were rinsed in PBS two times. The MTT solution was diluted with MEM medium to 0.5 mg/mL, and 1 mL of the solution was added to each well of a 24-well plate, followed by incubation in the dark for 2 h. After 2 h, the 24-well culture plate was taken out, all the liquid was carefully removed, 1 mL DMSO was added, the pipetted liquid was blown and mixed well, 200 μL was taken from each well and added to a new 96-well culture plate, three parallel multiple wells were set, and the OD at 470 nm was measured with the multifunctional marker.

***ALP***

Osteogenic differentiation of the preosteoblasts in each sample was investigated with alkaline phosphatase (ALP). Briefly, preosteoblasts were seeded on each specimen at a density of 5 × 10^4^ cells/mL (1 mL/well). After 24 h, the medium was replaced with OM. After incubation for 14 days, the cells were stained with the ALP Staining Kit (C3206, Beyotime, China) and quantified using ALP Assay Kit (P0321, Beyotime, China). The OD values for absorbance of the eluent were determined at 590 nm.

***Micro-CT and histopathological evaluation of osteogenesis***

After 6 weeks, the remaining rats were sacrificed and the left femurs with implants were harvested (n = 5). The tissues were fixed in 4% paraformaldehyde for 6 hours, and then Micro-CT was performed, of which the scanning accuracy was 20μm. The implant segment in the femur was chosen for the Region of interest (ROI) to analyzing data and reconstructing 3D images. Micro-CT analysis software (SCANCO Medical AG, Swiss) was used to calculate bone volume fraction (BV/TV) and the mean trabecular thickness (Tb.Th) of ROI.

After the assessment of Micro-CT, the implants were removed from the bones, and all the bone tissues were soaked in EDTA demineralization solution in 37℃, and in the shaker at a speed of 200rpm for two weeks. Subsequently, the tissues were fixed with paraffin wax, and then sectioned, dewaxed and washed in PBS. After staining with hematoxylin-eosin and van gieson, sections were dehydrated through increasing concentrations of ethanol and xylene.

***^1^H NMR spectrum, particle size testing, and Zeta potential***

The purified lyophilized complex PD, PAMAM, and DMADDM were sent to the Analysis and Test Center of Sichuan University for ^1^H NMR spectrum detection. Malvern Zetasizer Nano ZS was used to detect particle size and Zeta potential. The brief steps were as follows: PD, PAMAM, and DMADDM were dissolved in sterile deionized water, and mixed well to form a final concentration of 1mg/mL and added to the sample pool; the corresponding program for detection was selected; and each reagent was tested three times.

***Hemolysis test***

Reagents with different concentrations were configured: 100 mg/mL PAMAM/PD, 50 mg/mL PAMAM/PD, 25 mg/mL PAMAM/PD, 20 mg/mL PAMAM/PD, 10 mg/mL PAMAM/PD, and 5 mg/mL PAMAM/PD; PBS acted as the negative control and 10% Triton X-100 as the positive control. Sheep blood was added to the PBS solution, centrifuged at 500 × *g* for 5 min, and then resuspended with PBS, centrifuged, and purified at the top. After the blood cells were resuspended in PBS, 180 μL diluted blood cells were added to the 96-well culture plate at the bottom of the circle, followed by 20 μL solution to be measured, mixed well, and then cultured in a cell incubator (37℃, 5% CO_2_) for 1 h. After 1 h, the culture plate was centrifuged at 500 × *g* for 5 min and photographed. Subsequently, 100 μL was taken out from each well and added to the flat bottom 96-well culture plate, and the OD at 540 nm was measured with a multifunctional marker. Hemolysis rate = [sample OD (540 nm) − negative control OD (540 nm)]/ [positive control (OD 540 nm) − negative control (OD 540nm)].

| **Table S1 Primer sequences** | | |
| --- | --- | --- |
| Primer | Forward primers | Reverse primers |
| *alp* | CTGGCACAAAAGAGTTGGTAAGGC | GATCGGAACGTCAATTAACGTCAA |
| *ost* | TTTACAGCCTGCACCCAGATCC | TGCCCTTTCCGTTGTTGTCC |
| *dmp1* | CGGCTGGTGGACTCTCTAAG | CGGGGTCGTCGCTCTGCATC |
| *B2M* | TGCTATCCAGAAAACCCCTCAA | GCGGGTGGAACTGTGTTACG |

**Table S2** **The ARRIVE Guidelines Checklist**

| **ARRIVE Essential 10** | | | |
| --- | --- | --- | --- |
|  | ITEM | RECOMMENDATION | Section/Paragraph |
| Study design | 1 | For each experiment, provide brief details of study design including: a. The groups being compared, including control groups. If no control group has been used, the rationale should be stated. b. The experimental unit (e.g., a single animal, litter, or cage of animals). | 1. We have control groups in our study, Section 2.4 2. The experimental unit was a single animal, Section 2.4 |
| Sample size | 2 | a. Specify the exact number of experimental units allocated to each group, and the total number in each experiment. Also indicate the total number of animals used. b. Explain how the sample size was decided. Provide details of any a priori sample size calculation, if done. | a. Mentioned in Section 2.4  b. Mentioned in Section 2.4 |
| Inclusion and exclusion criteria | 3 | a. Describe any criteria used for including and excluding animals (or experimental units) during the experiment, and data points during the analysis. Specify if these criteria were established a priori. If no criteria were set, state this explicitly. b. For each experimental group, report any animals, experimental units, or data points not included in the analysis and explain why If there were no exclusions, state so. c. For each analysis, report the exact value of n in each experimental group. | a. Showed in Section 2.4.  b. Section 3.1.3  c. See in Section 2.4. |
| Randomisation | 4 | a. State whether randomisation was used to allocate experimental units to control and treatment groups. If done, provide the method used to generate the randomisation sequence. b. Describe the strategy used to minimise potential confounders such as the order of treatments and measurements, or animal/cage location. If confounders were not controlled, state this explicitly. | Section 2.4 |
| Blinding | 5 | Describe who was aware of the group allocation at the different stages of the experiment (during the allocation, the conduct of the experiment, the outcome assessment, and the data analysis). | Section 2.4, the second paragraph |
| Outcome measures | 6 | a. Clearly define all outcome measures assessed (e.g., cell death, molecular markers, or behavioural changes). b. For hypothesis-testing studies, specify the primary outcome measure, i.e., the outcome measure that was used to determine the sample size. | Section 2.4, Section3.1.3, Section 3.3 |
| Statistical methods | 7 | a. Provide details of the statistical methods used for each analysis, including software used. b. Describe any methods used to assess whether the data met the assumptions of the statistical approach, and what was done if the assumptions were not met. | a. Section 2.5, Supplementary materials  b. Section 2.5, Supplementary materials |
| Experimental animals | 8 | a. Provide species-appropriate details of the animals used, including species, strain and substrain, sex, age or developmental stage, and, if relevant, weight. b. Provide further relevant information on the provenance of animals, health/ immune status, genetic modification status, genotype, and any previous procedures. | a. Section 2.4  b. Section 2.4 |
| Experimental procedures | 9 | For each experimental group, including controls, describe the procedures in enough detail to allow others to replicate them, including: a. What was done, how it was done, and what was used. b. When and how often. c. Where (including detail of any acclimatisation periods). d. Why (provide rationale for procedures). | See in Section 2.4 |
| Results | 10 | For each experiment conducted, including independent replications, report: a. Summary/descriptive statistics for each experimental group, with a measure of variability where applicable (e.g., mean and SD, or median and range). b. If applicable, the effect size with a confidence interval. | Section 3.1.3 and Section 3.3 |
| **Recommended Set** | | | |
|  | ITEM | RECOMMENDATION | Section/Paragraph |
| Abstract | 11 | Provide an accurate summary of the research objectives, animal species, strain and sex, key methods, principal findings, and study conclusions. | In the Abstract Section |
| Background | 12 | a. Include sufficient scientific background to understand the rationale and context for the study, and explain the experimental approach. b. Explain how the animal species and model used address the scientific objectives and, where appropriate, the relevance to human biology. | See in the Section 1: Introduction |
| Objectives | 13 | Clearly describe the research question, research objectives and, where appropriate, specific hypotheses being tested. | Abstract;  Section 1;  Section 3: the first paragraph |
| Ethical statement | 14 | Provide the name of the ethical review committee or equivalent that has approved the use of animals in this study, and any relevant licence or protocol numbers (if applicable). If ethical approval was not sought or granted, provide a justification. | Section 2.4 |
| Housing and husbandry | 15 | Provide details of housing and husbandry conditions, including any environmental enrichment. | Section 2.4, the second paragraph |
| Animal care and monitoring | 16 | a. Describe any interventions or steps taken in the experimental protocols to reduce pain, suffering, and distress. b. Report any expected or unexpected adverse events. c. Describe the humane endpoints established for the study, the signs that were monitored, and the frequency of monitoring. If the study did not have humane end points, state this. | Section 2.4, the second paragraph |
| Interpretation/scientific implications | 17 | a. Interpret the results, taking into account the study objectives and hypotheses, current theory, and other relevant studies in the literature. b. Comment on the study limitations, including potential sources of bias, limitations ofthe animal model, and imprecision associated with the results. | Section 3.1.3, Section 3.3 and Section 3.5 |
| Generalisability/translation | 18 | Comment on whether, and how, the findings of this study are likely to generalise to other species or experimental conditions, including any relevance to human biology (where appropriate) | Section 3.1.3, the first paragraph |
| Protocol registration | 19 | Provide a statement indicating whether a protocol (including the research question, key design features, and analysis plan) was prepared before the study, and if and where this protocol was registered. | Section 2.4. |
| Data access | 20 | Provide a statement describing if and where study data are available. | Section 3 and Supplementary materials |
| Declaration of interests | 21 | a. Declare any potential conflicts of interest, including financial and nonfinancial. If none exist, this should be stated. b. List all funding sources (including grant identifier) and the role ofthe funder(s) in the design, analysis, and reporting ofthe study. | The part of Conflict of Interest and the Acknowledgement. |

**Results:**


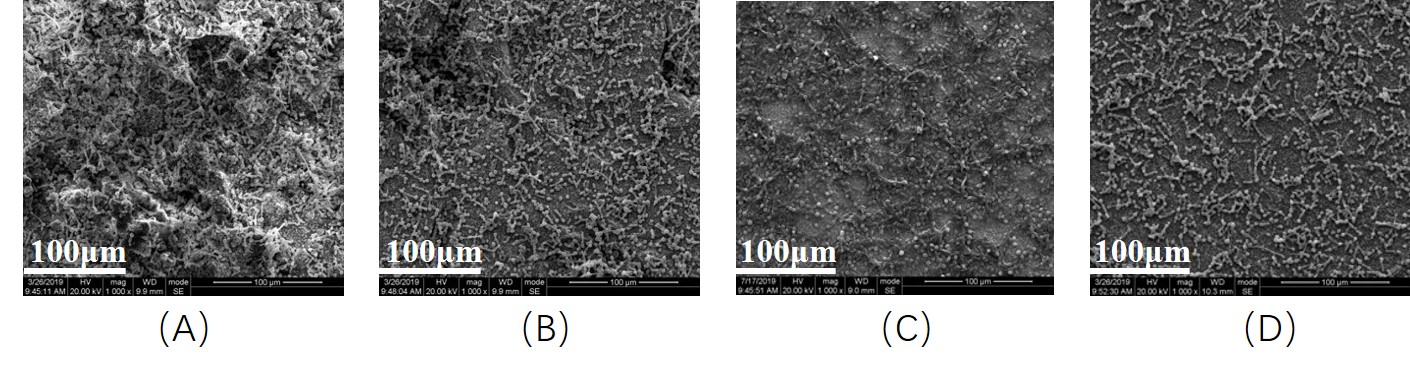

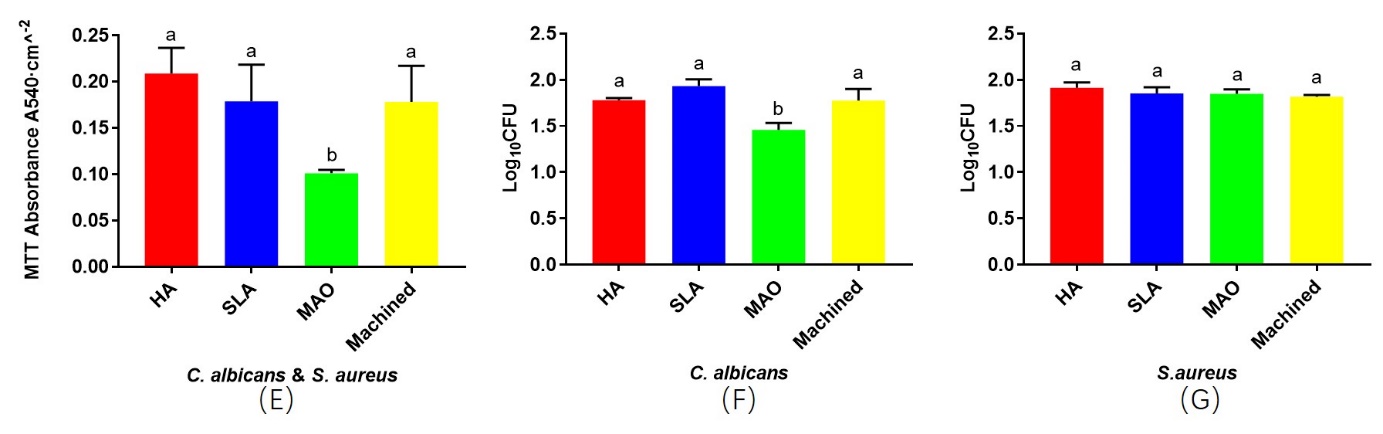


**Supplementary Figure 1 The antibacterial effect of the MAO implant. The structure of biofilms was observed via SEM: (A) HA plasma spraying; (B) SLA; (C) MAO treatment; (D) Machined titanium treatment; (E) biofilm metabolism; (F) & (G) CFU counts. The different letters indicate the significant difference between the bars (a, b).**


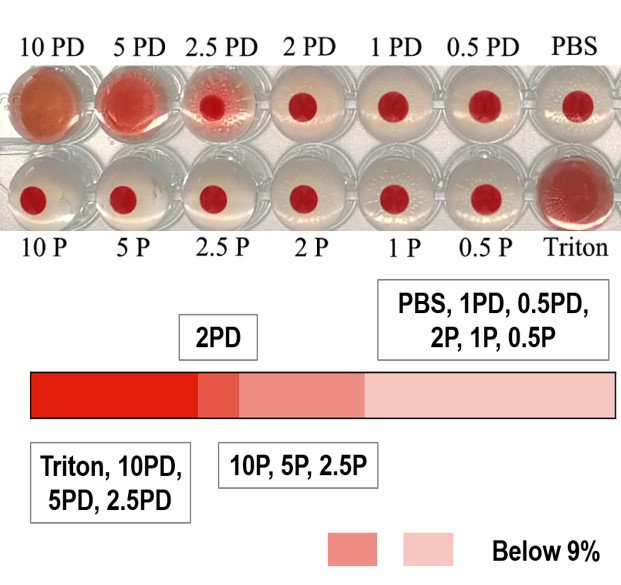


**Supplementary Figure 2 Hemolysis test (10 P, 5 P, 2.5 P, 2 P, 1 P, and 0.5 P are 10 mg/mL PAMAM, 5 mg/mL PAMAM, 2.5 mg/mL PAMAM, 2 mg/mL PAMAM, 1 mg/mL PAMAM, and 0.5 mg/mL PAMAM individually; 10 PD, 5 PD, 2.5 PD, 2 PD, 1 PD, and 0.5 PD are 10 mg/mL PD, 5 mg/mL PD, 2.5 mg/mL PD, 2 mg/mL PD, 1**

**mg/mL PD, and 0.5 mg/mL PD)**


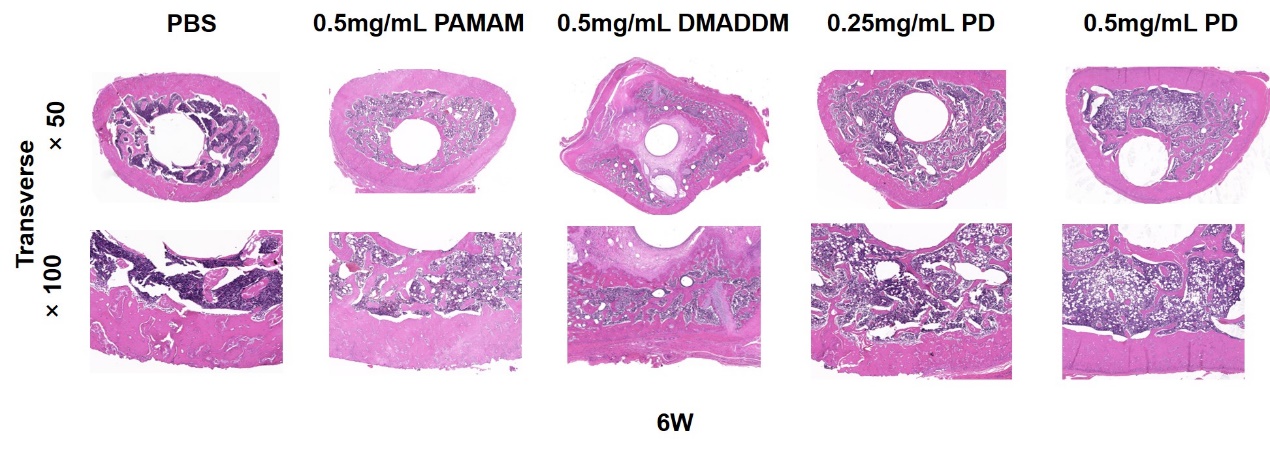


**Supplementary Figure 3 Histological images of transverse sections from a femur stained with hematoxylin and eosin at 6 weeks**

**
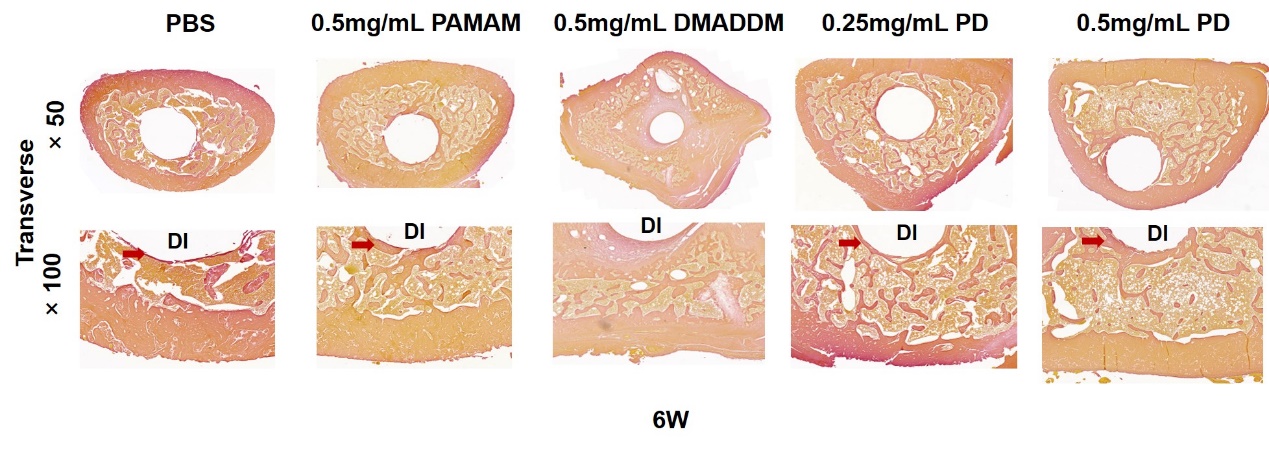
**

**Supplementary Figure 4 Histological images of transverse sections from a femur stained with Van Gieson stain at 6 weeks. DI is dental implant; red arrow points the new bone tissue stained red.**

**
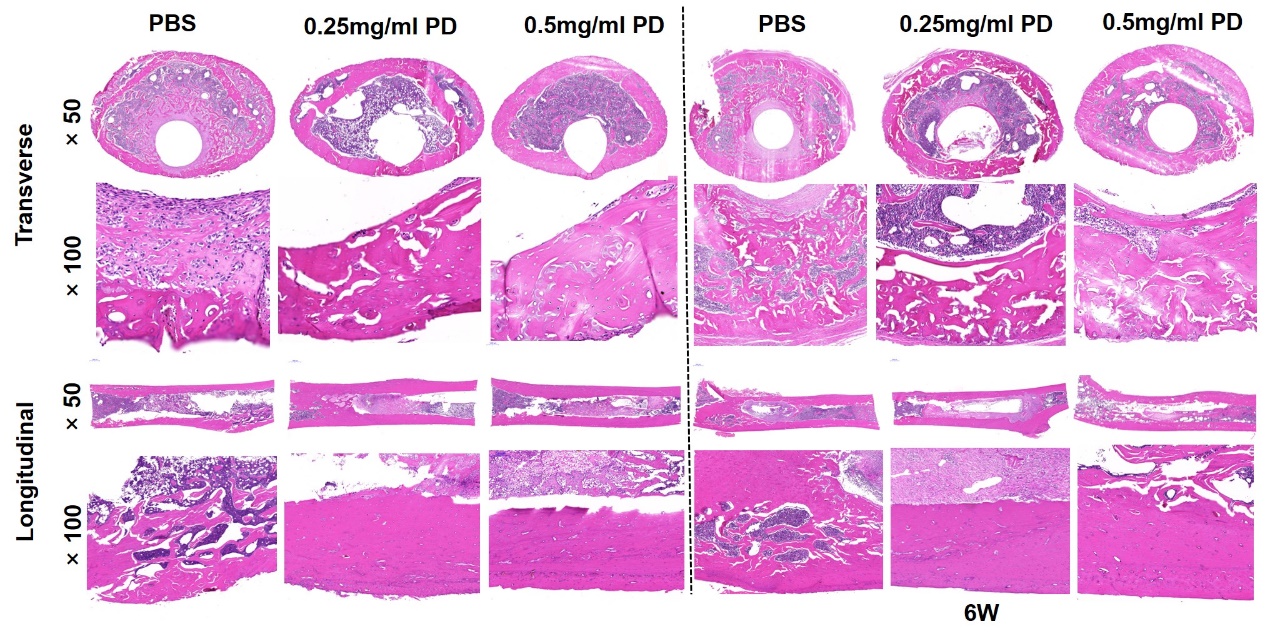
**

**Supplementary Figure 5 Effect of PD on osteogenic differentiation *in vivo*: HE staining**


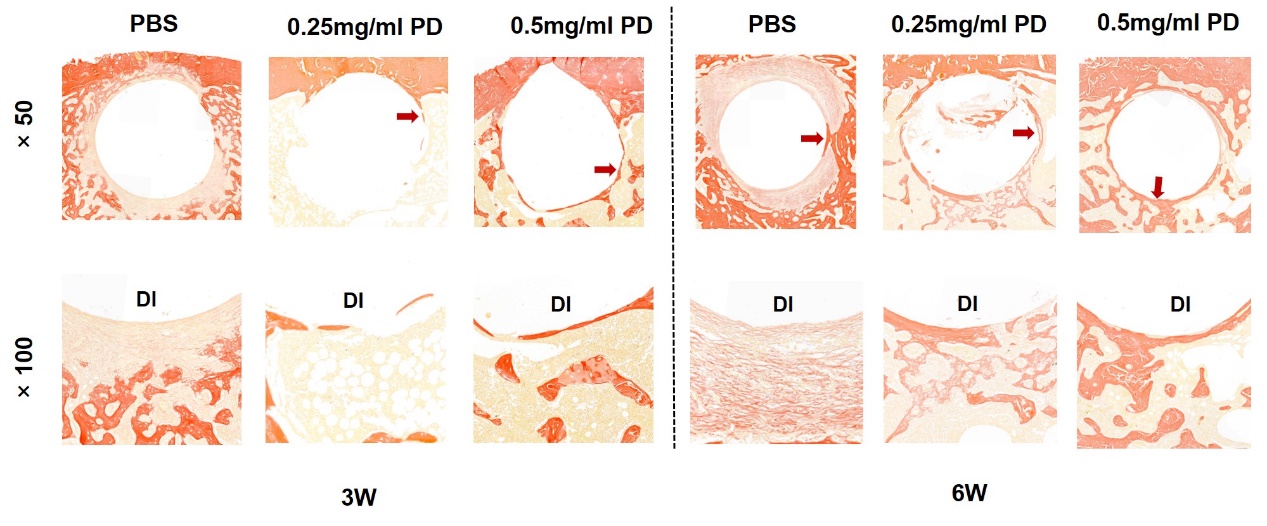


**Supplementary Figure 6 Effect of PD on osteogenic differentiation *in vivo*: van Gieson stain at 6 weeks. DI is dental implant; red arrow points the new bone tissue stained red.**

[1] X. Huang, W. Zhou, X.D. Zhou, Y. Hu, P. Xiang, B. Li, B. Yang, X. Peng, M. Li, L. Cheng, Effect of Novel Micro-Arc Oxidation Implant Material on Preventing Peri-Implantitis, Coatings. 9(11) (2019) 691. <https://doi.org/10.3390/coatings9110691>.

[2] B. Li, Y. Ge, Y. Wu, J. Chen, H.H.K. Xu, M. Yang, M. Li, B. Ren, M. Feng, M.D. Weir, X. Peng, L. Cheng, X. Zhou, Anti-Bacteria and Microecosystem-Regulating Effects of Dental Implant Coated with Dimethylaminododecyl Methacrylate, Molecules. 22(11) (2017) 2013. <https://doi.org/10.3390/molecules22112013>.

[3] X. Zhou, S. Wang, X. Peng, Y. Hu, B. Ren, M. Li, L. Hao, M. Feng, L. Cheng, X. Zhou, Effects of water and microbial-based aging on the performance of three dental restorative materials, J Mech Behav Biomed Mater 80 (2018) 42-50. <https://doi.org/10.1016/j.jmbbm.2018.01.023>.

[4] Y. Zhou, M. Liao, C. Zhu, Y. Hu, T. Tong, X. Peng, M. Li, M. Feng, L. Cheng, B. Ren, X. Zhou, ERG3 and ERG11 genes are critical for the pathogenesis of Candida albicans during the oral mucosal infection, Int J Oral Sci. 10(2) (2018) 9. <https://doi.org/10.1038/s41368-018-0013-2>.

[5] W. Zhou, X. Peng, Y. Ma, Y. Hu, Y. Wu, F. Lan, M.D. Weir, M. Li, B. Ren, T.W. Oates, H.H.K. Xu, X. Zhou, L. Cheng, Two-staged time-dependent materials for the prevention of implant-related infections, Acta Biomater. (2019). <https://doi.org/10.1016/j.actbio.2019.10.023>.
